# Multiple effects govern endogenous retrovirus survival patterns in human gene introns

**DOI:** 10.1186/gb-2006-7-9-r86

**Published:** 2006-09-27

**Authors:** Louie N van de Lagemaat, Patrik Medstrand, Dixie L Mager

**Affiliations:** 1 Terry Fox Laboratory, BC Cancer Research Centre, 675 W 10th Avenue, Vancouver, BC, V5Z 1L3, Canada; 2 Department of Medical Genetics, University of British Columbia, BC, V6T 1Z3 Canada; 3 Department of Experimental Medical Sciences, Lund University, BMC B13, 221 84 Lund, Sweden

## Abstract

**Background:**

Endogenous retroviruses (ERVs) and solitary long terminal repeats (LTRs) have a significant antisense bias when located in gene introns, suggesting strong negative selective pressure on such elements oriented in the same transcriptional direction as the enclosing gene. It has been assumed that this bias reflects the presence of strong transcriptional regulatory signals within LTRs but little work has been done to investigate this phenomenon further.

**Results:**

In the analysis reported here, we found significant differences between individual human ERV families in their prevalence within genes and degree of antisense bias and show that, regardless of orientation, ERVs of most families are less likely to be found in introns than in intergenic regions. Examination of density profiles of ERVs across transcriptional units and the transcription signals present in the consensus ERVs suggests the importance of splice acceptor sites, in conjunction with splice donor and polyadenylation signals, as the major targets for selection against most families of ERVs/LTRs. Furthermore, analysis of annotated human mRNA splicing events involving ERV sequence revealed that the relatively young human ERVs (HERVs), HERV9 and HERV-K (HML-2), are involved in no human mRNA splicing events at all when oriented antisense to gene transcription, while elements in the sense direction in transcribed regions show considerable bias for use of strong splice sites.

**Conclusion:**

Our observations suggest suppression of splicing among young intronic ERVs oriented antisense to gene transcription, which may account for their reduced mutagenicity and higher fixation rate in gene introns.

## Abstract 2

An analysis of human endogenous retrovirus families suggests suppression of splicing among young intronic retroviruses oriented antisense to gene transcription.

## Background

Transposable elements, including endogenous retroviruses (ERVs), have profoundly affected eukaryotic genomes [1-3]. Similar to exogenous retroviruses, ERV insertions can disrupt gene expression by causing aberrant splicing, premature polyadenylation, and oncogene activation, resulting in pathogenesis [4-6]. While ERV activity in modern humans has apparently ceased, about 10% of characterized mouse mutations are due to ERV insertions [5]. In rare cases, elements that become fixed in a population can provide enhancers [7], repressors [8], alternative promoters [9-11] and polyadenylation signals [12,13] to cellular genes due to transcriptional signals in their long terminal repeats (LTRs).

It has been previously shown that LTRs/ERVs fixed in gene introns are preferentially oriented antisense to the enclosing gene [14-16]. In contrast, *in vitro *studies of *de novo *retroviral insertions within gene introns in cell lines have not detected any bias in proviral orientation [17,18]. The fact that these integrations, which have not yet been tested for deleterious effect during organismal development, show no directional bias indicates that the retroviral integration machinery itself does not distinguish between DNA strands in transcribed regions. Presumably then, any orientation biases observed for endogenous retroviral elements must reflect the forces of selection. In support of this premise is a recent study by Bushman's group that was the first to directly compare genomic insertion patterns of exogenous avian leukosis virus after infection *in vitro *with patterns of fixed endogenous elements of the same family [17]. Endogenous elements in transcriptional units were four times more likely to be found antisense to the transcriptional direction, suggesting strong selection against avian leukosis virus in the sense direction. Therefore, the antisense bias exhibited by fixed ERVs/LTRs in genes suggests that retroviral elements found in the same transcriptional orientation within a gene are much more likely to have a negative effect. However, the mechanisms underlying these detrimental effects have not been analyzed in depth.

In this study, we explored the factors affecting the nascence of biases in ERV populations in genes. We began by demonstrating that the relative mutation frequencies in either orientation of an active family of mouse early transposon (ETn) ERVs account for directional bias of this family of elements in genes. Subsequent simulations of the activity of splice and polyadenylation signals contributed by these elements successfully accounted for the observed modes of transcriptional interference by intronic ETns. We further showed that the extent of antisense bias varies among human ERV (HERV) families and, correspondingly, that the predicted modes of transcriptional disruption of extant ERVs varied by family. This study highlighted the important role of splice sites in mutation, particularly splice acceptors, which allow for subsequent polyadenylation or splice donor usage. Evidence from human mRNAs demonstrated preferential usage of predicted strong splice sites occurring on either strand of ERV elements. However, splicing activity was found to be significantly down-regulated for antisense ERVs, especially younger ones. These observations suggest that splicing/exonization by antisense ERVs in introns is suppressed, perhaps due to hybridization with sense-oriented ERV mRNA, and may explain survival of antisense ERVs to fixation.

## Results

### Mutagenic ETn ERVs are oppositely oriented to overall genomic ETns

**Figure 1:**
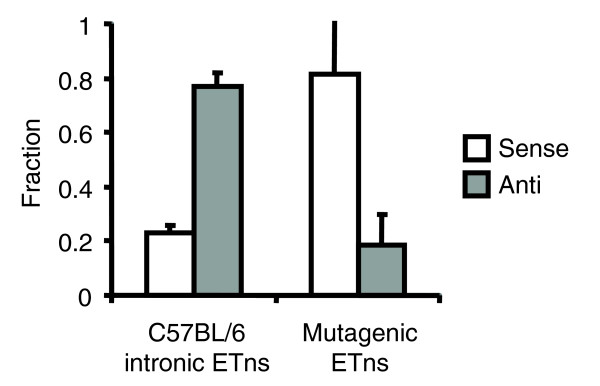
Directional bias of retroelements in mouse transcribed regions. ETn elements were those annotated as RLTRETN in the UCSC May 2004 mouse genome repeat annotation. The mutagenic population of ETn elements was reported in earlier reviews [5,19,20]. Expected variability in the data was calculated from Poisson statistics, which describe randomized gene resampling.

To begin our analysis of mechanisms contributing to ERV orientation bias, we reasoned that, if this bias is a consequence of detrimental impact by sense-oriented insertions, we would expect a predominant sense orientation among insertions with known detrimental effects. While no mutagenic or disease-causing ERV insertions are known in humans, significant numbers have been studied in the mouse and have been reviewed recently [5]. In particular, the ETn ERV family is currently active and causes mutations in inbred lines of mice. We therefore examined a recent data set of all published mouse ETn ERV mutations curated from the literature [5,19,20]. Of 18 mutagenic ETns within transcribed regions, 15 were in the same orientation as the enclosing gene and three were oriented antisense to gene transcription, in precise contrast to the annotated intronic ETn population present in the publicly available C57BL/6 genome (Figure 1) (see Materials and methods). This means that, while mutagenesis by antisense-oriented ETn elements is possible, sense-oriented mutagenesis is much more likely. Moreover, assuming ETn elements are representative of ERVs in general, these data suggest that, as expected, the orientation bias of ERVs is due to stronger negative selection against the more damaging sense-oriented intronic elements.

### Differences in antisense bias among families of fixed human ERVs

ERVs/LTR elements in the human genome actually comprise hundreds of distinct families of different ages and structures, many of which remain poorly characterized [21,22]. Thus, grouping such heterogeneous sequences together, as has been done for previous studies on orientation bias [15,16], may well mask variable genomic effects of distinct families. To investigate genic insertion patterns of different human ERV families, we chose nine Repbase-annotated [23] families or groups of related families with sufficient copy numbers to analyze in more detail. These families, their copy numbers and their approximate evolutionary time of first entry into the ancestral human genome are listed in Table 1. We required that ERVs in our study either be solely LTR sequence or contain both LTR and internal sequence in the same orientation within a 10 kb window (see Materials and methods).

**Figure 2:**
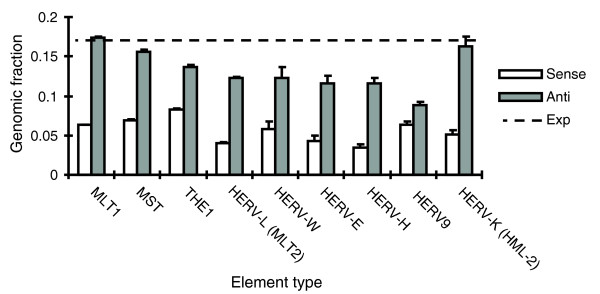
Orientation bias of various full length ERV sequences in genes. ERV families are as annotated by RepeatMasker in the human genome and are listed in Table 1. Fraction of all genomic elements actually found in genes in the sense and antisense orientations is presented, with neutral prediction (dotted line) based on fraction of total genomic elements expected in sense and antisense directions in genes under assumption of uniform random insertion.

We plotted the fraction of total genomic elements in either orientation found within maximal-length RefSeq [24] transcriptional units and the results are shown in Figure 2. Each family studied exhibited a bias for having more elements in the antisense direction to gene transcription. However, to put our results in a broader context, we considered a model of random initial integration throughout the genome. Since 34% of the sequenced genome falls within our analyzed set of RefSeq transcriptional units, we would expect 34% of ERV insertions, 17% in either direction, to be found in these regions. This is a conservative model since the initial integration patterns of most exogenous retroviruses are biased toward genic regions [17,18,25,26]. Relative to this model, many human ERV families exhibit significantly less antisense elements than expected by chance, and using Poisson statistics, which describe random sampling, we found that significant differences exist among the families in the relative prevalence of antisense elements (Figure 2). Similarly, there is significant variation among families in the genomic fraction of sense oriented elements retained in genic regions. However, relative to their antisense populations, most demonstrate a further two to threefold reduction in sense elements. The exception to this pattern was HERV9 (ERV9), which will be addressed further below.

### Significant variation in ERV antisense bias across transcriptional units

At least three factors could account for the antisense bias exhibited by most ERV families. First, the sense-oriented polyadenylation signal in the LTR could cause premature termination of transcripts and be subject to negative selection. Gene transcript termination within LTRs commonly occurs in ERV-induced mouse mutations [5] and this effect has been proposed as the most likely explanation for the orientation bias [16]. Second, paired splice signals within the interior of proviruses could induce exonization, a phenomenon also frequently observed in mouse mutations [5]. To address this second possibility, we plotted graphs similar to Figure 2 separately for solitary LTRs, which comprise the majority of retroviral elements in the genome [22,27], and for composite elements containing LTR and internal sequence (data not shown). Unfortunately, the numbers of the latter are much lower than for solitary LTRs for most families, making it difficult to detect significant differences in the density patterns. A third factor that could contribute to orientation bias is the potential of the LTR transcriptional promoter to cause ectopic expression of the gene, as occurs in cases of oncogene activation by retroviruses [6]. If introduction of an LTR promoter is a significant target of negative selection, one would predict that sense-oriented LTRs located just 5' or 3' to a gene's native promoter would be equally damaging and, therefore, subject to similar degrees of selection.

**Figure 3:**
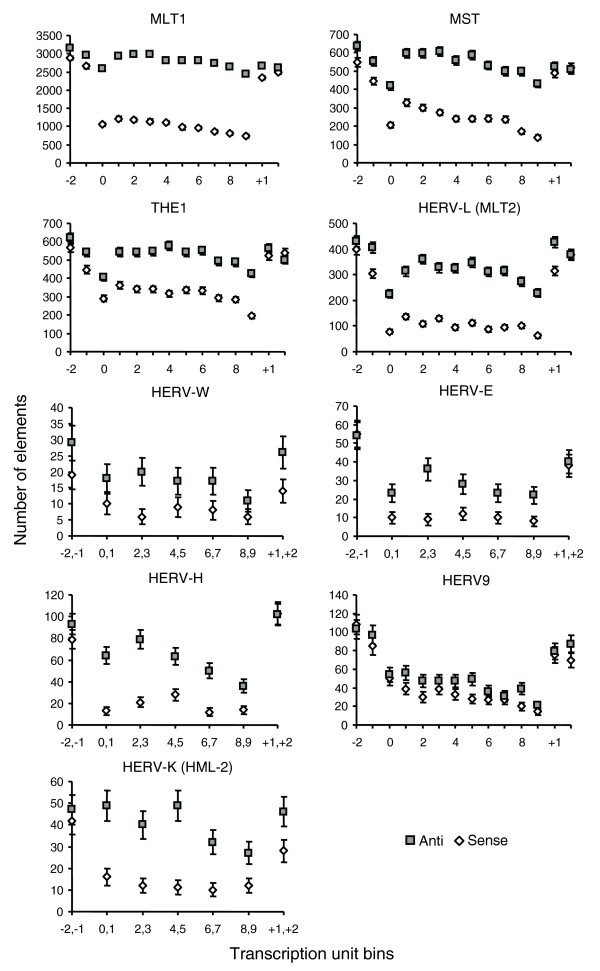
Numbers of annotated ERVs in equal-sized bins across transcriptional units. Ten bins, numbered 0 to 9, were considered within transcribed regions. Four bins, two in either direction outside gene borders and equal in length to intragenic bins, were considered, and are shown as bins -2 and -1 upstream and +1 and +2 downstream. For some ERV families, bins were combined to obtain sufficient numbers for analysis.

To gain deeper insight into the nature of orientation bias, we measured the absolute numbers of ERVs/LTRs of the same families in 10 bins, numbered 0 to 9, across the length of human RefSeq transcriptional units (Figure 3) (see Materials and methods). For comparison with transcribed regions, we included two bins of the same length upstream and downstream of each gene, numbered -2, -1, +1, and +2. This analysis revealed genic ERV density profiles that shift dramatically at gene borders. Specifically, for most ERV families, we found that the prevalence of sense-oriented elements drops markedly inside the 5' terminus of a gene, remains relatively low across the gene and then jumps just as markedly 3' of the gene. This deficit of sense-oriented elements accounts for the majority of the antisense bias of genic ERV populations.

Some ERVs, particularly HERV-L and the mammalian apparent LTR retrotransposons (MaLRs; MLT1, MST, and THE1), exhibited antisense bias upstream of transcriptional start sites, consistent with some degree of selection against their LTR promoter activity. However, the reduction in sense-oriented elements downstream of the gene's 5' terminus is, in most cases, greater than upstream of the start of transcription. Furthermore, the lack of sense-oriented elements persists across transcribed regions, which is more consistent with disruption of transcription in progress than with aberrant transcription initiation, although both factors could play a role.

**Figure 4:**
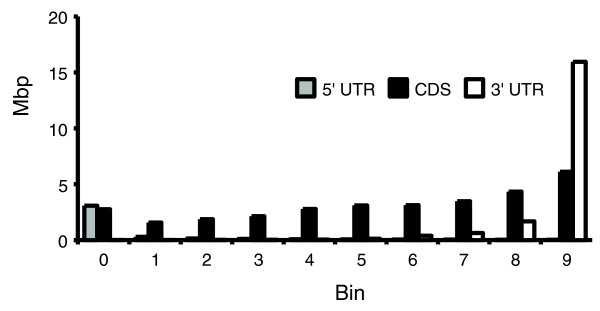
Total genomic sequence contributions by 5' untranslated regions (UTRs), coding sequences (CDSs), and 3' UTRs of RefSeq genes in transcription unit bins. Only transcripts corresponding to the longest transcribed region of each gene were considered.

Another feature notable in Figure 3 is that most ERV families exhibit a drop in density just inside transcription start sites (bin 0), followed by a higher density in the next internal bin. This observation is consistent with the fact that all first exons, as well as a significant amount of coding sequence, fall within bin 0 (Figure 4). Similarly, a low density of antisense ERVs in bin 9 is correlated with the presence of the terminal exons of genes and a significant amount of coding sequence (see Materials and methods). However, the observed reduction in element density by most antisense ERVs extended to the more central bins as well, with the expected negative correlation between the ERV density and coding sequence density.

### Sense-oriented splicing and polyadenylation signals of ETns predict mutations *in vivo*

**Figure 5:**
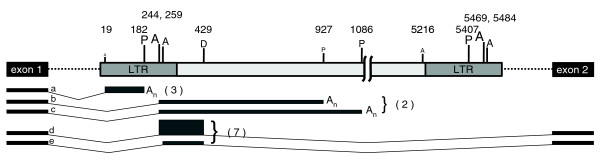
Analysis of an ETn ERV in the context of human *HOXA9*. A full length ETn ERV was placed in the context of *HOXA9 *intronic sequence and splice and polyadenylation signals were found using the programs GeneSplicer and polyadq, respectively (see Materials and methods). Signal strengths were determined by comparing software scores for each signal with profiles of signals found in human genes and are shown by their bar height and font size. P, polyadenylation signal; A, splice acceptor; D, splice donor. Base-pair position of each signal is shown above and is given in relation to the sequence of the ETn element used in this analysis (NCBI accession Y17106). The five most frequent events predicted by *in silico *transcription assay are lettered 'a' to 'e' and their relative frequencies are shown by the thickness of the predicted exons. These exons correspond to *in silico *exonizations 14, 8.4, 8.4, 36, and 8.0 percent of the time. Numbers in parentheses are actual cases of ETn-mediated transcriptional disruption [5].

The distinct distributions and orientation bias patterns of different ERV families (Figures 2 and 3) suggest that their intronic presence affects genes in distinct ways, presumably through the transcriptional regulatory signals they harbor. We therefore attempted to model the consequences of ERV insertions and began by using ETn elements as a test case. ETn elements typically cause mutations by disrupting splicing and/or polyadenylation of the enclosing gene and, in some cases, the aberrant transcripts have been molecularly characterized (for a review, see [5]). These data provided an opportunity to determine if we could predict the detrimental consequences of intronic insertion of a sense-oriented ERV element by conducting a computer simulation study. The publicly available programs GeneSplicer [28] and polyadq [29] were used to profile splicing and polyadenylation scores of all human genes. We then used the same programs and the human genic profiles to calculate likelihood of usage of splicing and polyadenylation signals found within a full-length ETn element when placed within an intron of the human *HOXA9 *gene (see Materials and methods). We chose a fully-sequenced mutagenic ETn element (NCBI Accession number Y17106) that is highly similar to most other known cases of ETn mutations [5]. Repeat-free sequence from the intron of the *HOXA9 *gene provided genomic upstream and downstream sequence for the element, allowing discovery of transcriptional signals in the first and last 100 base-pairs (bp) of the ERV. In this analysis, we considered an ERV 'mutagenic' if it supplied both the upstream splice acceptor (SA) site and the downstream splice donor (SD) or polyadenylation signal. A bootstrapping analysis involving 10,000 simulated transcriptions across this field of probabilistic splice donor and acceptor sites was performed, resulting in an array of predictions of transcription disruption of the enclosing gene (Figure 5; Additional data file 1). Bootstrap trials were terminated once an exonization was calculated to have occurred.

Modes of transcriptional interference events identified by our bootstrapping analysis involved use of cryptic SA sites in the ETn element followed by downstream termination by polyadenylation or splicing out using a SD site. The most frequent mode of transcriptional interference predicted was an exonization event that accounted for 36% of all simulated transcription. This exonization involved a SA site found within the 5' LTR downstream of the natural polyadenylation site and a SD site within the ERV internal region (event d in Figure 5). An additional 17% of simulated transcripts involved the same SA site but terminated at one of two closely spaced cryptic polyadenylation signals downstream of the SD site (events b and c). A third high-frequency event involved a SA site in the U3 region of the 5' LTR and subsequent polyadenylation at the natural LTR polyadenylation signal (event a). This event accounted for 14% of simulated transcription. This analysis accurately recapitulates the most frequent modes of transcriptional disruption curated from the literature by Maksakova and colleagues [5] (Figure 5). It is worth noting that both documented, *in vivo *transcriptional disruptions and predicted splicing events are biased to relatively upstream splice sites, suggesting that our *in silico *transcription approach is indeed realistic.

Unexpectedly, analysis of the ETn sequence in the antisense direction predicted similar frequencies of transcriptional disruption. However, individual splicing and polyadenylation signals were much less strong, leading to a large number of low-frequency predicted modes of transcriptional disruption (Additional data file 1). Similarly to ETns in the sense orientation, the predicted events involved both internal exonization and premature polyadenylation. Potential explanations for this unanticipated finding are examined below.

### Transcriptional signals of sense-oriented ERVs suggest variation in modes of transcriptional disruption among ERVs

**Figure 6:**
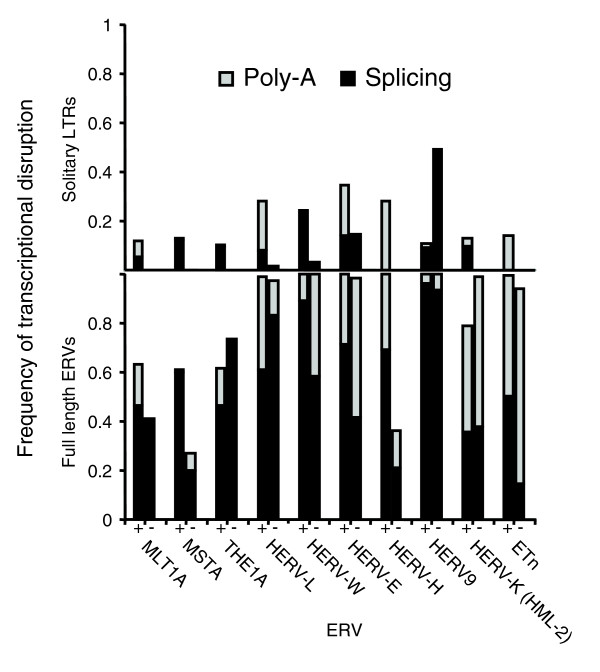
*In silico *transcriptional disruption frequencies for full length ERVs and related solitary LTRs. ERV consensus elements in either orientation were placed in the context of the human *HOXA9 *gene and probabilities of usage of splice sites and polyadenylation signals were computed (see Materials and methods). An *in silico *bootstrapping technique was used to estimate overall frequencies of transcriptional disruption due to these signals. Two bars are shown for each ERV type in each panel, with bars on the left-hand sides representing modes of transcriptional disruption for ERVs in the sense direction, and data for antisense elements in the right-hand side bars. The upper and lower panels represent disruption frequencies by solitary LTRs and full length ERVs, respectively. Grey bars represent polyadenylation events (for example, events 'a' to 'c' in Figure 5) and black bars correspond to fully spliced exonization events (for example, events 'd' and 'e' in Figure 5).

Given our success in predicting the major known modes of transcriptional disruption by sense-oriented ETn elements, we extended the analysis to human ERVs, in this case using sequences of consensus ERV elements (see Materials and methods). This analysis revealed that, while premature polyadenylation is predicted to be a prominent form of transcript disruption, especially for HERV-K elements, polyadenylation alone does not explain all mutagenesis by sense-oriented ERVs (Figure 6). Rather, similar to the ETn case, splicing leading to internal exonization also likely plays an important role in ERV-mediated mutagenesis, especially for the HERV-W and HERV9 elements. This analysis also demonstrated a much greater propensity for transcriptional disruption by full-length elements compared to solitary LTRs in every case. Furthermore, similar to the ETn case, predicted transcriptional disruption events were biased to splice sites encountered early in transcription through ERV proviral structures. Additional checking of sense-oriented ERVs revealed additional strong splice sites downstream of dominant transcription disruption events, but due to our bootstrapping technique, these often remained unused (data not shown). Finally, similar to ETn ERVs, and as discussed below, analysis of the antisense strand of consensus human ERVs revealed similar numbers of splice and polyadenylation motifs, resulting in predicted high probability of transcript disruption by antisense ERVs in genic regions (Figure 6).

One relevant caveat is that this analysis was performed to condense a large number of individual signal likelihoods spread over the consensus ERV elements into a unified prediction of transcriptional disruption. Therefore, no checks were done on the predicted exon size, with the result that 7% of the total predicted exons have an SA-SD distance or SA-polyadenylation signal distance of a size smaller than the first percentile length of exons of human genes (39 or 91 bp, respectively; data not shown). Although this minority of predicted exons may not be biologically significant, they nevertheless illustrate the activity of the splice sites and polyadenylation signals they employ.

### ERV9s cause transcription disruption in the sense and antisense direction

As mentioned above, we found the orientation bias patterns of ERV9 within transcribed regions especially intriguing. Within genic regions, ERV9 antisense bias was the least among all ERV families studied (Figure 2). The extension of this analysis in ten bins across transcribed regions (Figure 3) showed that this low bias persisted all across transcribed regions. We therefore re-examined projected transcriptional interference patterns mediated by ERV9 (Figure 6) and found strong exonization activity in both orientations. In the sense orientation, this activity was concentrated in the internal region, with 83% of simulated transcription disrupted by spliced exons with both splice sites entirely within the ERV internal region (Additional data file 1). In contrast, the predicted activity of antisense ERV9s is prominently associated with splice sites in the LTR, with 49% of simulated transcription disrupted by fully spliced exons within a solitary LTR, which was represented in our analysis by the RepBase LTR12C consensus. By comparison, a full-length antisense ERV9 is projected to disrupt gene transcription 100% of the time (see Figure 6). This likelihood of transcriptional disruption in the antisense direction by solitary ERV9 LTRs may explain the decreased prevalence of antisense elements within transcribed regions.

### Activity of splicing signals in ERV internal regions is confirmed by mRNA evidence but absent in young, antisense ERVs

As mentioned above, analysis of ERV sequences suggests a much greater propensity for transcriptional disruption by full-length elements than solitary LTRs, an effect associated with promiscuous splice acceptor sites in full length elements. Furthermore, our computer simulation method predicts a similar degree of transcriptional disruption for both strands of many of the ERVs examined (Figure 6). However, the higher prevalence of antisense-oriented ERVs in genic regions suggests that they are generally less damaging to genes than those oriented in the same direction. One explanation for our results is simply that the modeling method is not accurate and gives more weight to splice or polyadenylation sites that are not functional and/or predicts a much higher level of transcription disruption than would actually occur *in vivo*. Alternatively, we considered the possibility that splicing is down-regulated in some way for antisense ERVs, drastically reducing their propensity to transcriptional disruption until fixation.

To determine if the predicted splicing signals on both strands of ERVs were actually used, we conducted an analysis of human mRNAs and the repeat annotation from the May 2004 University of California Santa Cruz (UCSC) Genome Browser [30]. For simplicity, and given the importance of splice acceptor sites, we restricted our analysis of transcriptionally active signals to splice sites. Splice sites with multiple mRNA support that mapped within the internal part of full-length ERV structures were recorded (see Materials and methods). Then, 100 bp of genomic sequence flanking the splice site was aligned to the appropriate ERV consensus to determine the base pair position of the splice site within the consensus ERV.

**Figure 7:**
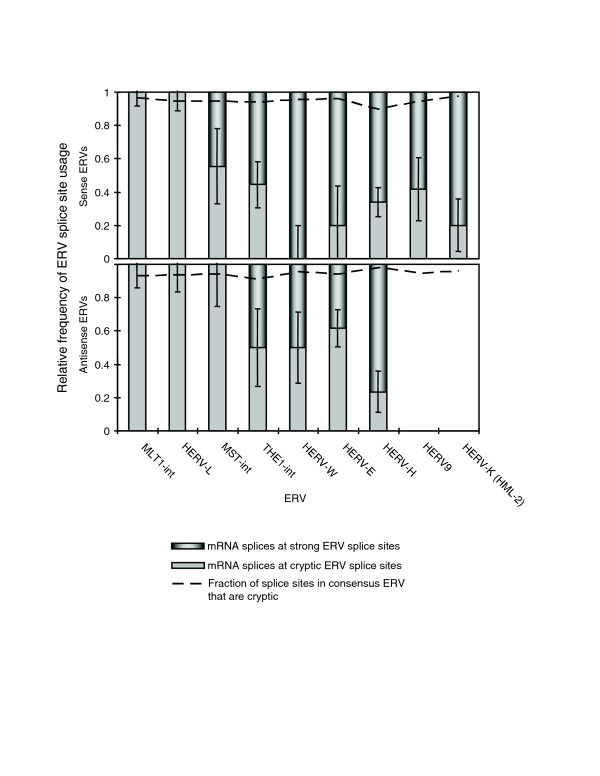
Association of splice sites in human mRNAs with strong and cryptic splice sites identified in full-length ERVs. Upper and lower panels are for sense and antisense ERVs, respectively. ERVs are shown in approximate order of origin or most recent activity. Dashed lines represent the fraction of simple AG and GT splice site motifs in the consensus ERV that are cryptic. Variability indicated is calculated by Poisson statistics. HERV-L is represented by four consensus elements (see Materials and methods). Old ERVs, such as MLT1 and HERV-L, exhibit splicing exclusively at cryptic splice sites. mRNA splicing within younger elements, such as THE1A, HERV-E, and HERV-H, is found at both strong and cryptic sites. The recently active ERVs, HERV9 and HERV-K (HML-2), show no splicing activity at either strong or cryptic splice sites when found in the antisense direction in introns, while these ERVs demonstrate significant splicing activity when found in the sense direction.

We then used our mRNA splice event data to assess the frequencies with which annotated splicing events coincided with positions of predicted strong ERV splice motifs. For purposes of this analysis, we considered sites identified by GeneSplicer on either strand of the ERV consensus as 'predicted', and other sites with the basic GT and AG motifs as 'cryptic'. In the case of no preference for strong splice sites, we would expect the observed mRNA splice events to associate with cryptic and predicted splicing motifs in proportion to their relative abundances in the consensus element. We found that old ERVs, particularly the older MLT1 MaLR and HERV-L elements, did indeed match this expectation (Figure 7, Additional data file 2), while younger ERVs, such as HERV-E and HERV-H, demonstrated highly significant bias for usage of predicted splice sites. This observation held for both sense and antisense ERVs.

The splicing behavior of antisense HERV9 and HERV-K (HML-2) elements was most puzzling. For these relatively young proviruses, predicted and cryptic splicing motifs occur with similar frequency on both strands (Additional data file 2). However, in contrast to 12 and 10 splicing events found in human mRNAs in the sense orientation, respectively, no splicing events were detected by our method in the antisense direction. This is despite the fact that more antisense elements are found within genes, providing more opportunity to engage in gene splicing. This difference is significant (p < 0.01 in both cases, calculated from the binomial distribution).

## Discussion

We have conducted an analysis of factors involved in nascence of orientation bias among families of endogenous retroviral-like elements in the human genome. As a first step, our reanalysis of data on characterized mutagenic ETn insertions confirmed that mutation frequency in either orientation precisely accounts for the directional bias of the surviving ETn genic population in the mouse genome. This study also documented considerable variation in antisense bias among different human ERV families. At the most basic level, this observation indicates that each ERV is a distinct entity with a distinct transcriptional disruption profile. In addition, however, we found that many families of ERVs exhibit less antisense elements in genic regions than expected from a purely random insertion model. It seems reasonable that, of the many ERV families that have infected the germ line over the course of evolution, the significant correlation between integration in genes and mutagenicity results in a decreased likelihood for ERVs that target genes to survive to fixation in a species. This may explain the general observation that most of the ERV families that have reached high copy numbers in the primate lineage, exemplified by the ERVs studied, have less members in transcribed regions, even in the antisense direction, than expected by random chance. An alternative explanation might be that differing propensity among ERVs to disrupt coding sequence results in a greater or lesser loss of antisense elements. For example, there is an obvious negative correlation between the prevalence of antisense MLT1 elements across genic regions and the likelihood of disruption of coding exons (Figures 3 and 4).

Analysis of populations of sense and antisense oriented elements across transcriptional units showed that antisense orientation bias is dominated by an abrupt decrease in the sense oriented population of elements coincident with the start of transcription, and a similar abrupt increase downstream of the transcribed region. The fact that some sense oriented ERVs do persist may be a reflection of early partial or complete deletion of internal sequence either by random deletion or recombination between the 5' and 3' LTRs, removing strong splicing signals that are necessary for mutagenic splicing and polyadenylation events to occur.

As a means to gain further insight into mutagenesis by ERVs, *ab initio *splice site and polyadenylation signal prediction methods were first used to analyze the sequence of an active ETn element in the genomic context of a human gene (*HOXA9*) and succeeded in identifying the highest-frequency transcriptional disruption modes reported in studies of ETn-induced mutations [5,19,20]. This analysis clearly illustrated the necessity for a functional SA site as a prerequisite for mutagenesis by exonization or premature polyadenylation. Moreover, the success in predicting ETn-induced transcriptional disruption suggested the feasibility of this method for prediction of mutagenesis modes of human ERVs, in this case using consensus ERV sequences that presumably reflect the original sequence of these elements at the time of insertion.

Analysis of ETn and human ERV sequences by this method revealed two primary findings. The first is that full-length elements have a much higher potential to cause mutagenesis compared to solitary LTRs. This is perhaps not surprising, since functional retroviruses and ERVs contain splice signals that direct transcription of the various transcripts in the proportions required for successful protein translation and correct assembly of viral particles. A second, initially unexpected trend also became apparent. We found it surprising that splicing and polyadenylation motifs within antisense ERVs were, on the whole, similar in strength to those on the sense strand. Indeed, the number of ERV families suggested by this analysis to cause transcriptional disruption more than 95% of the time was similar in both directions. This result led to an examination of actual instances of ERV transcriptional signal usage in forming human mRNAs. This survey revealed that older ERVs, such as MLT1A and HERVL, exhibited splicing only at cryptic sites, whereas younger ERVs, such as HERV-E and HERV-H, were strongly skewed to use of predicted splice sites. These findings confirm that splice sites predicted on both strands of the ERV are indeed potentially active and sites predicted in antisense ERVs are not simply an artifact of the prediction program. Furthermore, this result suggests slow loss of the original, canonical splice sites over evolutionary time, with other cryptic sites evolving at random locations.

**Figure 8:**
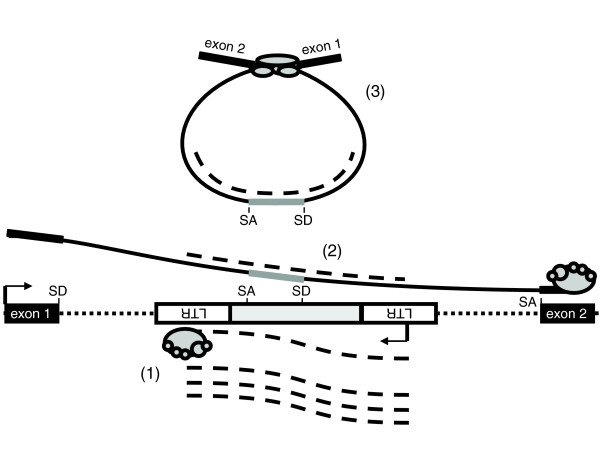
Model of antisense ERV retention in introns of cellular genes. Sense oriented ERV mRNA, shown by dashed lines, is transcribed from the LTR promoter (1). During transcription of the cellular gene, ERV mRNA anneals to the nascent cellular mRNA, shown as a solid line (2). During splicing, annealed ERV mRNA sterically hinders access to splice sites within the antisense ERV sequence, repressing exonization (3).

In light of predictability of mutagenic events evidenced by the ETn family, as well as mRNA confirmation of the existence of splicing motifs on both ERV strands, it puzzled us that many ERV elements are allowed to persist in the antisense direction in spite of their splice signal strength. One potential explanation for this situation comes from the observation of the complete lack of splicing activity by antisense HERV9 and HERV-K elements, while these same elements do exhibit splicing in the sense direction. This effect is consistent with antisense-mediated redirection of splicing (for a review, see [31]). It has been shown that antisense RNA directed either against splice signals or motifs entirely within exons can result in exon skipping. Furthermore, RNA complementary to splice signals has resulted in exon skipping as well, due to masking of the splice signals. We propose that a similar phenomenon has allowed a greater fraction of antisense ERVs to survive to fixation (Figure 8). In this model, transcripts of the intronic ERV, which is oriented antisense to gene transcription, or transcripts from similar ERV elements elsewhere in the genome, can anneal to nascent pre-mRNA being transcribed from the gene's sense strand. In support of this model, persistent genic ETn elements are predominantly found in the antisense direction and, while mostly expressed early in embryogenesis, also demonstrate low levels of transcription in most cell types studied [5] (unpublished observations). A similar splicing suppression effect, directed against exons of human genes, has been postulated as a potential therapy for Duchenne Muscular Dystrophy [31].

It seems conceivable that, at least early after insertion, this effect could control transcriptional disruption by antisense ERVs. We conjecture that continuation of this suppression over longer evolutionary times may be achieved by selection for low-level transcription of these elements. However, more detailed analysis, including cell based assays, is required before we can pinpoint the precise source of such potential interfering RNA.

As an alternative to, or in addition to, splicing suppression by antisense RNA, deletions of key splice sites, either by small deletions within the internal region or by recombination between the flanking LTRs, may account for a reduced likelihood of mutation compared with that of the consensus element and thus partially explain genomic tolerance of antisense ERVs in genic regions. For example, it has been appreciated for some time that HERV-H has reached high copy number in primate genomes in a deleted form, termed RTVL-H [32]. In that case, the consensus full-length elements we have analyzed represent, numerically, only a minor variant that has enabled much more successful deleted forms to propagate through the host genome. Nevertheless, long term usage of potent splice signals on both strands of ERVs, as evidenced by our survey of human mRNAs, suggests that this mechanism can only partially, if at all, explain antisense bias in genic regions.

## Conclusion

Analysis of factors involved in nascence of orientation bias has revealed several interesting findings, ultimately suggesting a complete model for mutagenesis by sense-oriented genic ERVs and concomitant toleration of most antisense ERV insertions. First, our analysis demonstrated that human ERV families differ significantly from one another, both in terms of overall prevalence in genic regions and in their orientation bias. Furthermore, significant variation was observed in ERV orientation bias patterns across transcribed regions, consistent with this hypothesis. Secondly, software analysis of splicing and polyadenylation signals contained in mouse ERVs demonstrated the feasibility of prediction of the mode of transcriptional disruption of each ERV. Extension of this analysis to human ERVs demonstrated that full length ERVs are most mutagenic, due to internal strong splice sites contained in ERV internal regions. This analysis also illustrated the critical importance of the splice acceptor site in initiating a transcriptionally disruptive event, and the sufficiency of either splice donor or polyadenylation signals for completion of the event. Finally, evidence from human mRNA splicing patterns within internal regions of ERVs strongly suggested a mechanism of splicing suppression, likely by steric hindrance of splicing within full length antisense ERVs due to annealing of sense oriented ERV mRNAs. This mechanism can explain the increased tolerance of genic regions to antisense insertions. Over longer evolutionary times, loss of key splice sites by point mutation and deletion of ERV internal sequence likely obviates the requirement for this suppression. These observations have the potential to explain the pervasive pan-species antisense bias exhibited by ERV retroelements.

## Materials and methods

### Directional bias of insertions in transcribed regions in mice

Retroelement and gene annotation from the UCSC April 2004 C57BL/6 Mouse Genome Browser [30] was used to assess insertion frequency and orientation of insertions within the longest RefSeq transcribed regions of mouse genes. ETn LTR elements were represented by the RLTRETN family of ETn/MusD LTRs, and pairs of elements within 10 kb of each other and in the same orientation were assumed to belong to the same original insertion. The antisense bias observed in the C57BL/6 genic ETn LTR population was then compared to genic orientation bias in a data set of documented mutagenic ETn/MusD LTR insertions from earlier studies [5,19,20].

### Variation of antisense bias among human ERV families and across transcriptional units

RepeatMasker [33] annotations of endogenous retroviral elements from the human May 2004 UCSC Genome Browser [30] were compiled and compared to annotated transcribed region start and end points of the per-chromosome longest transcription unit of each RefSeq gene, defined by its HUGO gene name, to study orientation bias in transcribed regions.

Full length ERVs were identified by matching RepBase [23] annotations for internal elements with those of their respective LTRs as follows: HERVE or Harlequin internal sequences were matched with LTR2, LTR2B, or LTR2C; HERVK (HML-2) with LTR5, LTR5_Hs, LTR5A, or LTR5B; HERV17 with LTR17 (where HERV17 represents HERV-W); HERVH with LTR7, LTR7A, or LTR7B; HERV9 with LTR12, LTR12B, LTR12C, LTR12D, LTR12E, or LTR12_; *ERVL* (where * represents a wildcard) with MLT2*; MST* with MST*-int; THE1* with THE1*-int; and MLT1* with MLT1*-int. Groups of LTR element segments of the same type, with internal sequence all in the same orientation, and occurring within a 10 kb window were deemed part of the same composite element. Manual checks confirmed the validity of this criterion. Names of consensus elements occurring in each composite element were recorded, as well as names of the ERV type. Composite elements without internal sequence were deemed LTR-only, and elements with contributions from at least two consensus elements were deemed to contain LTR and internal sequence and, therefore, were considered full-length. Again, manual checking confirmed the validity of this criterion. These data were used in the construction of Table 1, and Figures 2 and 3.

For purposes of Figure 2, the assigned genomic position of each LTR or full-length element was computed as the average of the beginning and end coordinates of each composite element and compared against the positions of the longest transcription units of each gene. Each transcribed region was treated as a single bin for comparison of orientation bias among ERV families. For purposes of Figure 3, transcriptional units were divided into ten equal bins for analysis of orientation bias across transcription units, and ERV location within a gene was specified by which of the bins its average position fell into. In addition to the ten intragenic bins, two bins upstream and two bins downstream of the gene, of the same size as the intragenic bins, were also considered. Counts of elements for each orientation were computed for each bin.

### Internal and terminal exon forming capacity of ERVs

Mappings of RefSeq genes to the May 2004 human genome were downloaded from the UCSC Genome Browser. A nonredundant set of human genomic splice acceptor and splice donor sites was obtained for exons of RefSeq genes. The corresponding sequence for each locus, including 200 bp upstream and downstream, was then obtained and splice site strength evaluated by the publicly available program GeneSplicer [28]. Splice sites identified in our RefSeq set also identified by GeneSplicer were profiled and separate score probability distributions were constructed representing all used splice acceptors and donors.

By a similar method, a nonredundant set of terminal exons was constructed for human RefSeq genes and characterized with respect to each terminating polyadenylation signal. Genomic sequence of the mapped terminal exons, including 200 bp flanking regions upstream and downstream, was obtained, and the publicly available program polyadq [29] was used to evaluate polyadenylation signal strengths. The polyadenylation signal prior to the mapped polyadenylation site was taken as that used in transcription. Manual checking confirmed that this site was, in general, the highest-scoring motif anywhere in the sequence considered, suggesting the sufficiency of this criterion in identifying the correct polyadenylation signal. Again, a probabilistic software score distribution was constructed representing polyadenylation signals in human genes.

Probabilistic identification of exons in ERVs was performed by placing a consensus ERV element within the context of a human gene intron. For this purpose, we chose the human *HOXA9 *locus with approximately 2.5 kb flanking both upstream and downstream of the gene. This gene was chosen because it has a single transcript with a single short (approximately 1 kb) intron, is easily identified by computational gene finders, and occurs in a repeat-free region.

With the exception of the ETn element analyzed (a fully-sequenced element, NCBI Accession number Y17106), synthetic ERV elements were constructed from an internal consensus with two related flanking consensus LTRs. MaLR elements in humans have been described as consisting of nine total subfamilies based on LTR sequence [34]; however, the number of defined Repbase consensus LTRs is much higher. The number of actual subfamilies of internal sequence is likely of the same order as the number of LTR subfamilies, although only six internal MaLR consensus sequences are defined in Repbase [23]. Therefore MaLRs were represented by the MLT-int consensus paired with MLT1A1 LTRs, MST-int with MSTA LTRs, and THE1-int with THE1A LTRs. Other ERV consensus elements analyzed included HERV17 with LTR17 LTRs (which represents HERV-W), HERVE with LTR2, HERVH with LTR7B, HERV9 with LTR12C, and HERVK with LTR5_Hs LTRs (representing the HML-2 family of HERV-K). Given the large number of HERV-L families, we attempted to address some of this variability by constructing four consensus HERV-L elements that use MLT2-family LTRs, and amalgamating the results. The four consensus elements consisted of the ERVL-B4 internal consensus with its related MLT2B4 LTRs, the ERVL-E internal element with MLT2E LTRs, HERVL with MLT2A1 LTRs, and ERVL with its related MLT2B1 LTRs. Ambiguity codes in each consensus ERV element were replaced with corresponding unambiguous nucleotide letters drawn from aligning genomic elements.

Use of flanking genomic sequence allows GeneSplicer and polyadq to identify splice and polyadenylation signals near the ends of the constructs. Therefore, and as mentioned above, the ETn or synthetic ERV constructs were placed in the context of the *HOXA9 *intron at position 4,000 of the construct, corresponding to position 506 of the intron. GeneSplicer and polyadq were used to identify theoretically useful splice and polyadenylation signals within the ERV. A simple bootstrapping method using 10,000 simulated transcriptions over this probabilistic signal field was then used to assess the likelihood of exonization involving internal ERV sequence. The software score distributions calculated from human gene splice sites were used to evaluate the likelihood of usage of each signal. Predicted short exons with less than 39 bp between SA and SD sites or less than 91 bp between SA and polyadenylation signal were permitted, but formed a small minority of exons predicted.

### Analysis of mRNA splice events within ERV internal elements

We used human transposable element and mRNA annotations from the May 2004 UCSC Genome Browser. The best mapping of each mRNA, determined by identity to the genome, was parsed for locations of intron-exon boundaries. We required that each intron-exon boundary be confirmed by at least two mRNAs. We therefore also parsed mappings of ESTs to the genome to gain additional evidence for splice sites obtained from the mRNA mappings. Thus, EST evidence was only used to confirm splice sites found in the mRNA database, but not to contribute additional splice sites.

As before, we chose the Repbase internal consensus elements MLT1-int, MST-int, THE1-int, HERV17 (corresponding to HERV-W), HERVE, HERVH, HERV9, and HERVK. HERV-L was represented by the ERVL, ERVL-B4, ERVL-E, and HERVL internal consensus elements, and the results from these elements were lumped in later analyses. ERV structures that were broken up, either by intervening repeats or other sequence, were joined if they belonged to the same consensus, were in the same orientation, and were found within a 10 kb window. The ERV sequence within each structure was required to be at least 2 kb long. The mammalian apparent LTR elements, MLT1-int, MST-int, and THE1-int, which have a shorter consensus 1 to 2 kb long, were required to be at least 500 bp in length.

Intron-exon boundaries that mapped within these ERV internal regions were aligned to the appropriate consensus internal element to map the physiological splice site to its location in the consensus element. These mappings were then compared to the locations of splice sites predicted by the program GeneSplicer [28].

### Coding sequence effects

We used the RefSeq transcript mappings to the May 2004 human UCSC Genome Browser to find the longest transcribed region of each gene and catalogued the number of genomic base pairs that map to the untranslated regions and coding exons of each transcript. Longest transcribed regions were divided into 10 bins, as before, and the total amount of sequence belonging to each exon, or exon fragment, was assigned to a bin based on the center position of the sequence fragment.

## Additional data files

The following additional data are available with the online version of this paper. Additional data file 1 provides complete *in silico *ERV splicing prediction results. Additional data file  2 contains results of analysis of genomic mRNA splicing events within ERVs.

## Supplementary Material

Additional data file 1 Complete *in silico *ERV splicing prediction results Click here for file

Additional data file 2 Results of analysis of genomic mRNA splicing events within ERVs Click here for file

## Figures and Tables

**Table 1:** Genomic annotated ERV structures and evolutionary ages of various ERV families

Name	Total copy number*	Full length^†^	Evolutionary age of origin (Mya)	Reference^‡^
MLT1	160 k	36 k	>100	[34]
MST	34 k	5,175	75	[34]
THE1	37 k	9,019	55	[34]
HERV-L (MLT2)	25 k	4,777	>80	[35]
HERV-W	675	242	40-55	[36]
HERV-E	1,138	294	25	[37]
HERV-H	2,508	1,284	>40	[38]
HERV9	4,837	697	15	[39]
HERV-K (HML2)	1,206	178	30	[40, 41]

*Including LTRs with no internal sequence and LTRs with associated internal sequence (see Materials and methods). ^†^Elements including both LTR and internal sequence. ^‡^Representative references with descriptions of each ERV family. Mya, million years ago.
